# Hederasaponin C Alleviates Lipopolysaccharide-Induced Acute Lung Injury *In Vivo* and *In Vitro* Through the PIP2/NF-κB/NLRP3 Signaling Pathway

**DOI:** 10.3389/fimmu.2022.846384

**Published:** 2022-02-25

**Authors:** Shan Han, Renyikun Yuan, Yushun Cui, Jia He, Qin-Qin Wang, Youqiong Zhuo, Shilin Yang, Hongwei Gao

**Affiliations:** ^1^ College of Pharmacy, Guangxi University of Chinese Medicine, Nanning, China; ^2^ State Key Laboratory of Innovative Drug and Efficient Energy-Saving Pharmaceutical Equipment, Jiangxi University of Traditional Chinese Medicine, Nanchang, China; ^3^ Guangxi Engineering Technology Research Center of Advantage Chinese Patent Drug and Ethnic Drug Development, Guangxi University of Chinese Medicine, Nanning, China

**Keywords:** acute lung injury, hederasaponin C, epigenetic, PIP2, NF-κB, NLRP3 inflammasome

## Abstract

Gene transcription is governed by epigenetic regulation that is essential for the pro-inflammatory mediators surge following pathological triggers. Acute lung injury (ALI) is driven by pro-inflammatory cytokines produced by the innate immune system, which involves the nod-like receptor 3 (NLRP3) inflammasome and nuclear factor-κB (NF-κB) pathways. These two pathways are interconnected and share a common inducer the phosphatidylinositol 4,5-bisphosphate (PIP2), an epigenetic regulator of (Ribosomal ribonucleic acid (rRNA) gene transcription, to regulate inflammation by the direct inhibition of NF-κB phosphorylation and NLRP3 inflammasome activation. Herein, we report that hederasaponin C (HSC) exerted a therapeutic effect against ALI through the regulation of the PIP2/NF-κB/NLRP3 signaling pathway. In lipopolysaccharide (LPS)/lipopolysaccharide + adenosine triphosphate (LPS+ATP)-stimulated macrophages, our results showed that HSC remarkably inhibited the secretion of interleukin-6 (IL-6), IL-1β, and tumor necrosis factor-α (TNF-α). Moreover, HSC inhibited NF-κB/p65 nuclear translocation and the binding of PIP2 to transforming growth factor-β activated kinase 1 (TAK1). The intracellular calcium (Ca^2+^) level was decreased by HSC *via* the PIP2 signaling pathway, which subsequently inhibited the activation of NLRP3 inflammasome. HSC markedly alleviated LPS-induced ALI, restored lung function of mice, and rescued ALI-induced mice death. In addition, HSC significantly reduced the level of white blood cells (WBC), neutrophils, and lymphocytes, as well as pro-inflammatory mediators like IL-6, IL-1β, and TNF-α. Hematoxylin and eosin (H&E) staining results suggested HSC has a significant therapeutic effect on lung injury of mice. Interestingly, the PIP2/NF-κB/NLRP3 signaling pathway was further confirmed by the treatment of HSC with ALI, which is consistent with the treatment of HSC with LPS/LPS+ATP-stimulated macrophages. Overall, our findings revealed that HSC demonstrated significant anti-inflammatory activity through modulating the PIP2/NF-κB/NLRP3 axis *in vitro* and *in vivo*, suggesting that HSC is a potential therapeutic agent for the clinical treatment of ALI.

## Introduction

SARS-CoV-2 is a novel RNA β-coronavirus causing a pandemic infection of coronavirus disease 2019 (COVID-2019) (1). When SARS-CoV-2 infects an organism, immunological and inflammatory responses, as well as cytokine storms, occur, resulting in lung diseases like pneumonia, acute respiratory distress syndrome, or even acute lung injury (ALI) (2). ALI is characterized by endothelial damage to the pulmonary capillaries and alveolar epithelial lesions, refractory hypoxemia, and non-cardiac acute pulmonary edema (3). It is generally caused by uncontrolled innate immunity and leads to the malfunction of the lung alveolar-capillary membrane barrier, growing production of pro-inflammatory cytokines, and infiltration of neutrophils and other immune cells (4, 5). The endotoxin lipopolysaccharide (LPS) is a well-known inducer of ALI in mice. By activating innate immune cells, LPS has been demonstrated to induce the production of pro-inflammatory cytokines and chemotactic molecules, ultimately resulting in lung tissue damage (6). Previous studies indicated that pro-inflammatory cytokines in the bronchoalveolar lavage fluid (BALF) have a more pronounced effect than those in serum in ALI (7). Generally, the release of pro-inflammatory cytokines, such as interleukin-6 (IL-6), interleukin-1β (IL-1β), and tumor necrosis factor-α (TNF-α) are highly released in the serum and BALF, which further regulate and promote the progress of ALI (8). Therefore, suppressing the aberrant release of pro-inflammatory cytokines is an effective strategy to treat acute lung injury in clinical.

Phosphatidylinositol-4,5-bisphosphate (PIP2), an epigenetic regulator of Ribosomal ribonucleic acid (rRNA) gene transcription, regulates cell membrane dynamics, cell shape, differentiation, proliferation, ion channels, and transporters (9). A previous study showed that ALI was also related to abnormal gene expression patterns caused by an epigenetic process involving the PIP2 signaling pathway (10). PIP2 lipids interact directly with TAK1 at W241 and N245 and promote its activation, which activates the NF-κB/NLRP3 inflammasome signaling pathway in the course of ALI (11, 12). NLRP3 inflammasome is primed with NLRP3, ASC, and caspase-1, which responds to a range of initiating stimuli. Upon activation of NLRP3 inflammasome, cleaved caspase-1 and mature IL-1β are formed to induce different cellular damages (13). The NF-κB signaling pathway may stimulate the synthesis of the NLRP3 inflammasome. Then, IL-1β formation might be facilitated and secreted extracellularly (14). Several studies indicated that Ca^2+^ mobilization is important for NLRP3 inflammasome activation (15). Ca^2+^ release from the endoplasmic reticulum into mitochondria triggers mitochondrial Ca^2+^ overload and damage (16). PIP2 is hydrolyzed to inositol-1,4,5-trisphosphate (IP3) and diacylglycerol (DAG) upon phospholipase C (PLC) activation. IP3 involves in the intracellular Ca^2+^ release process. DAG stimulates PKC to adjust local signaling (17). Therefore, to suppress the NF-κB signaling and NLRP3 inflammasome activation through PIP2 is an effective strategy to decrease pro-inflammatory cytokines release, which could alleviate ALI.

Hederasaponin C (HSC), a bioactive triterpenoid saponin, is the major active constituent of *Pulsatilla chinensis* (Bunge) Regel, which has been widely used in traditional Chinese medicine. Hederasaponin C showed considerable antischistosomal activity and a therapeutic effect on colitis (18, 19). However, the detailed mechanism and therapeutic effect of HSC on ALI were not found. Thus, the present study aimed to investigate the therapeutic effect of HSC and its underlying mechanism on ALI *in vitro* and *in vivo*.

## Materials and Methods

### Reagents

Hederasaponin C (>98%) was isolated from *Pulsatilla chinensis* (Bunge) Regel in our laboratory and determined by HLPC. (4,5-Dimethylthiazol-2-yl)-2,5-diphenyltetrazolium bromide (MTT), LPS (Escherichia coli, serotype 0111: B4), Ca^2+^ detector of Fluo-3/AM, were purchased from Sigma-Aldrich (St. Louis, MO, USA). PierceTM Protein A/G Magmetic Beads (#88802) were obtained from Thermo Fisher Scientific. Dulbecco’s modified eagle medium (DMEM), 1640, fetal bovine serum (FBS) were acquired from Gibco (Grand Island, NY, USA). ATP were purchased from Macklin (Shanghai, China). IL-1β (#224603-014), IL-6 (#225266-022), and TNF-α (#234889-001) ELISA kits were obtained from Invitrogen (Vienna, Austria). Antibodies against PLCγ2(#3872, western blotting:1:1000), IP3 Receptor1 (#8568, western blotting:1:1000), DAG Lipaα (#13626, western blotting:1:1000), TAK1 (#4505, western blotting:1:1000), p-TAK1 (#9339, western blotting:1:1000), PKCα (#2056S, western blotting:1:1000), p- PKCα (#9375S, western blotting:1:1000), p65 (#8242T, western blotting:1:1000), p-p65 (#3033, western blotting:1:1000), poly (ADP-ribose) polymerase (PARP) (#9532, western blotting:1:1000), NLRP3 (#15101, western blotting:1:1000), ASC (#13833, western blotting:1:1000), IL-1β (#31202, western blotting:1:1000), Cleaved-Caspase-1 (#89332 and #4199S, western blotting:1:1000), Cleaved-IL-1β (#63124 and #83186S, western blotting:1:1000), GAPDH (#5174, western blotting:1:1000) and the secondary antibodies including anti-rabbit IgG, HRP-linked Antibody (#7074) and anti-mouse IgG, HRP-linked Antibody (#7076) were obtained from Cell Signaling Technology (Beverly, MA, USA). Caspase-1 (ab1872, western blotting:1:1000) were obtained from Abcam (Cambridge, MA, USA). PIP2 (#53412, western blotting:1:1000) were obtained from Senta (Santa Cruz Biotechnology, USA).

### Cell Culture

The Cell Bank of the Chinese Academy of Sciences (Shanghai, China) provided RAW264.7 and THP-1 cells, the Kunming Cell Bank of the Chinese Academy of Sciences (Kunming, China) provided J774A.1 cells. RAW264.7 and J774A.1 cells were grown in DMEM (10% FBS). THP-1 cells were cultured in RPMI 1640 medium (10% FBS). All cells were cultured in a CO_2_ concentration of 5% with a temperature of 37°C.

### Cell Viability Assay

RAW264.7 (4×10^4^ cells/well), J774A.1 (2×10^4^ cells/well), and THP-1 (2×10^4^ cells/well) were plated into 96-well plates overnight. The cells were pro-treated with HSC (0, 20, 40, and 80 μM) for 24 h. Each well was added MTT solution and incubated for 4 h. The plate was detected by a microplate reader with an absorbance of 570 nm.

### Flow Cytometry

In 12-well plates, RAW264.7 (1.5×10^5^ cells per well), J774A.1 (1×10^5^ cells per well), and THP-1 (1×10^5^ cells per well) cells were seeded overnight. Following a 4 h pre-treatment with HSC (0, 20, 40, and 80 μM), the cells were co-treated with another 6 h with or without LPS (1 μg/ml). Fluo-3/AM (1 mM, 1 h) probes was used to measure the level of intracellular Ca^2+^. An automated flow cytometer (Becton-Dickinson, Franklin Lakes, NJ, USA) was used to detect the fluorescence intensity. For the analysis, 1×10^4^ cells were collected and completely gated.

### Enzyme-Linked Immunosorbent Assay (ELISA)

The concentration of IL-6 and TNF-α was determined by using ELISA kits according to the manufacturer’s specifications. In 12-well plates, RAW264.7 (1.5×10^5^ cells per well), J774A.1 (1×10^5^ cells per well), and THP-1 (1×10^5^ cells per well) cells were cultivated overnight. The cells were pre-processed with HSC (0, 20, 40, and 80 μM) for 4 h, then stimulated with LPS (1 μg/ml) for 18 h. The supernatant was collected and the concentration of IL-6 and TNF-α was measured by using an ELISA. The concentration of IL-1β was determined according to the manufacturer’s specifications. In 12-well plates, J774A.1 (1×10^5^ cells per well) and THP-1 (1×10^5^ cells per well) cells were grown overnight. The cells were pre-processed with HSC (0, 20, 40, and 80 μM) for 4 h, the cells were stimulated with LPS (1 μg/ml) for 6 h, and then co-treated with ATP (5 mM) for further 30 minutes. The supernatant was collected to detect the concentration of IL-1β.

### Live Cell Imaging

RAW264.7 and THP-1 cells were plated into 96-well plates at densities of 4×10^5^ and 2×10^5^cells per well, respectively overnight. The cells were pre-treated with HSC (80 μM) for 4 h before being co-cultured with or without LPS for another 6 h. The cells were labeled with Fluo-3/AM (10 μM) for 1 h. The fluorescence images were captured by a fluorescence microscope (Leica, DMi8, Wetzlar, Germany).

### Immunofluorescence

As previously mentioned, the immunofluorescence of NF-κB/p65, PIP2, PLCγ2, NLRP3, Caspase-1, ASC, and Ca^2+^ was investigated (20). Briefly, 2×10^5^ RAW264.7 cells were seeded into a confocal dish overnight (SPL, Pocheon, Korea). The cells were pre-treated with HSC (80 μM) for 4 h before being stimulated with LPS (1 μg/mL) for further 2 h. Incubation with anti-NF-κB/p65 antibody (1:100) overnight at 4°C, followed by incubation with goat anti-rabbit Alexa Fluor 594 secondary antibody (1:200) for 1 h at room temperature. After that, the cells were fixed and stained for 30 minutes with Hoechst 33342 (1 μM) before being imaged. The immunofluorescence of Caspase-1, PIP2, PLCγ2, and ASC was conducted using the same method as with NF-κB/p65, except the different dilutions of anti-Caspase-1 (1:200), anti-PIP2 (1:100), anti-PLCγ2 (1:100), anti-ASC (1:100). Goat anti-mouse IgG Fluor^®^ 488 Conjugate (#S0017, 1:200), Alexa Fluor 488 (1:200) secondary antibody. The cells were imaged with a confocal laser microscope (Leica, Wetzlar, Germany).

J774A.1 cells (3×10^5^ cells per well) were plated into 6-well plates overnight. Plasmids of EGFP-Ca^2+^ and EGFP-NLRP3 were transfected for 48 h by using turboFect transfection reagents (#R0531, Thermo Fisher Scientific, Grand Island, NY, United States). Then the cells were seeded in a confocal dish (SPL, Pocheon, Korea) overnight. The immunofluorescence of Ca^2+^ and NLRP3 was conducted by using the above method.

### Western Blotting Analysis

RAW264.7, J774A.1, and THP-1 cells were plated into a dish at a density of 1×10^6^ cells overnight. Pre-treatment with HSC (0, 20, 40, and 80 μM) for 4 h was followed by stimulation with LPS. RIPA lysis buffer was used to extract the proteins. The cytoplasmic and nuclear proteins were extracted by using a nuclear and cytoplasmic protein extraction kit (Beyond time, Shanghai, China) according to the manufacturer’s instructions. The quantities of proteins were measured using a BCA protein kit (Waltham, MA, USA).

The antigen sample was treated with the specified antibody overnight at 4°C with mixing for IP experiments, and then the protein A/G magnetic beads were washed and collected using a magnetic stand before being mixed with the antigen/antibody combination. It was necessary to wash the beads three times with IP buffer. To do western blot analysis, denatured proteins were separated using 8%, 10%, or 12% SDS-PAGE gels and transferred to a PVDF membrane (Millipore, Billerica, MA, USA). After 2 h of blocking with 5% non-fat milk, the PVDF membrane was incubated with antibodies against PIP2 (1:1000), PLCγ2 (1:1000), IP3 Receptor1 (1:1000), DAG Lipaα (1:1000), TAK1 (1:1000), p-TAK1 (1:1000), PKCα (1:1000), p- PKCα (1:1000), p65 (1:1000), p-p65 (1:1000), PARP (1:1000), NLRP3 (1:1000), ASC (1:1000), IL-1β (1:1000), Caspase-1 (1:1000), Cleaved-Caspase-1 (1:1000), Cleaved-IL-1β (1:1000), and GAPDH (1:1000) for more than 12 h at 4°C and secondary antibody anti-rabbit IgG, HRP-linked Antibody and anti-mouse IgG, HRP-linked Antibody (1:5000) for 2 h at room temperature. The protein band signals were detected using Super Signal West Femto maximum sensitivity substrate (Pierce Biotechnology, USA) under the help of ChemiDoc MP Imaging System (Bio-Rad, Hercules, CA, USA).

### Quantitative Real-Time PCR (qRT-PCR) Assay

J774A. 1 cells were pretreated for 4 h with HSC (20, 40, and 80 μM) and subsequently with LPS (1 μg/ml). Total RNA was extracted, and 1 g of RNA was tested using qRT-PCR. The PCR amplification process included SYBR green. TNF-α, IL-6, PLCγ2, PIP2, IP3 Receptor 1, DAG Lipase α and GAPDH oligonucleotide primers were as follows:

TNF-α-F: TTCTGTCTACTGAACTTCGGGGTGATCGGTCC,
TNF-α-R: GTATGAGATAGCAAATCGGCTGACGGTGTGGG,
IL-6-F: TCCAGTTGCCTTCTTGGGAC,
IL-6-R: GTGTAATTAAGCCTCCGACTTG,
PLCγ2-F: ACACTCTCCTTCTGGCGGTCTG,
PLCγ2-R: GGATGAGGGCGTAGATGCTGTTG,
PIP2-F: GACCTGTGCCTTCTGCCTTACG,
PIP2-R: CCCACTGTGCTTTGCTGAATITGAG,
IP3 Receptor 1-F: GTTTGAGAATTTCCTCGTGGAC,
IP3 Receptor 1-R: CATCACGATTTCAGTGACGTAC,
DAG Lipase α-F: TGCTAGGGAGGGTGCTATGTCTG,
DAG Lipase α-R: GTCCAATGGCAGTAACAGGTGTAGG,
GAPDH-F: TGCCTCCTGCACCACCAACT,
GAPDH-R: CCCCGTTCAGCTCAGGGATGA.

### Animals Experiments

This study was conducted under the approval of the Ethics Committee for Experimental Animal Management of Guangxi University of Chinese Medicine (NO. SYXK-GUI-2019-0001). All animals were well cared for in accordance with the Local Guidelines for the Care and Use of Laboratory Animals of Guangxi University of Chinese Medicine. Healthy BALB/c mice (male, 6-8 weeks old, weighing 18-22 g) were purchased from Hunan SJA Laboratory Animal Co. Ltd (Changsha, Hunan, China) and acclimated for 3 days. All animals were housed under standard specific pathogen-free (SPF) conditions and given free access to water and a safe diet at room temperature (20-26°C, daily temperature difference ≤4°C) and relative humidity (40%-70%).

#### LPS-Induced Acute Lung Injury of Mice

Mice were just free to water without food for 12 h before modeling. All of the mice were allocated into six groups at random according to their body weight. Intratracheal injection (i.t) was used to establish an ALI model as our previous study (21). Briefly, mice were injected with 0.4% sodium pentobarbital (i.p.) at a dose of 0.14 mL/10 g. After the mice were anesthetized, they were placed supine on the operating table, their mouths were opened with a mouse opener, and LPS (4 mg/kg or 15 mg/kg) (control group was injected with an equivalent amount of sterile saline) was injected into the lung through the opening of the epiglottis cartilage with a micro-nebulizer. The model was successfully established, then water and food were given after they woke up.

The following three kinds of animal experiments were conducted separately according to the above modeling method:

1. All mice were randomly assigned to six groups (n=10 per group): control group, model group, HSC intravenous injection (5, 10, and 20 mg/kg, i.v.), dexamethasone group (DEX, 5 mg/kg). In exclusion of the control group, the other groups received instillation of LPS (15 mg/kg, i.t.) into the lung of mice. Following LPS treatment, mice were treated with HSC (5, 10, and 20 mg/kg) at time points of 0, 12, 24, 48, and 72 h. While mice were treated with dexamethasone (i.p.) at 0 h after LPS treatment. The survival rate of mice within 156 h was observed.
2. All mice were randomly assigned to six groups (n=10 per group): control group, model group, HSC intravenous injection (5, 10, and 20 mg/kg, i.v.), dexamethasone group (DEX, 5 mg/kg). In exclusion of the control group, the other groups received instillation of LPS (4 mg/kg, i.t.) into the lungs of mice. Following LPS treatment, mice were treated with HSC (5, 10, and 20 mg/kg) at time points of 0 and 12 h. While mice were treated with dexamethasone (i.p.) at 0 h after LPS treatment. After 24 h, the lung respiratory function of mice was detected by using an AniRes2005 lung function test system (Bestlab, Beijing, China).
3. All mice were randomly assigned to six groups (n=12 per group): control, model, HSC intravenous injection (5, 10, and 20 mg/kg, i.v.), dexamethasone (5 mg/kg). In exclusion of the control group, the other groups received instillation of LPS (4 mg/kg, i.t.) into the lungs of mice. Following LPS treatment, mice were treated with HSC (5, 10, and 20 mg/kg) at time points of 0 and 12 h. While mice were treated with dexamethasone (i.p.) at 0 h after LPS treatment. Serum, bronchoalveolar lavage fluid (BALF; see below for BALF collection instructions), and lung tissue samples were obtained and preserved after 24 h. TNF-α, IL-6, and IL-1β ELISA kits were used to quantify cytokines in serum and BALF samples. For lung tissues, cytokine levels were determined using ELISA kits after lung tissue homogenates. For histological investigation, a piece of lung tissues was fixed in 4% paraformaldehyde, then embedded in paraffin. Western blotting was used to determine the protein level in lung tissues.

#### An Assessment of the Respiratory Function of Mice

The mice were anesthetized after 24 h of HSC treatment, and then the trachea was cut open and a 2 mm stiff casing was introduced and secured into the trachea. The catheter was connected to an AniRes2005 lung function test system (Bestlab, Beijing, China). The mice were mechanically ventilated at a rate of 90 breaths per minute with a tidal volume (VT) of 10 mL/kg, utilizing a water column to generate a positive end-expiratory pressure of 5 cm H_2_O. We measured lung resistance (RL), expiratory resistance (Re), and respiratory lung compliance (Cdyn).

#### Enzyme-Linked Immunosorbent (ELISA) Assay

After centrifuging the blood sample at 3000 rpm, 4°C for 20 minutes, serum samples were collected. Additionally, the BALF was collected and the supernatant was centrifuged at 1600 rpm for 10 minutes at 4°C. Lung tissues were homogenized using a tissue grinder (TP-24, Tianjin, China) and centrifuged at 3000 rpm and 4°C for 20 minutes. The supernatant was collected and kept at a temperature of -80°C. All samples were tested by using ELISA kits.

#### Histopathological Examination

The lung tissue samples were fixed with 4% paraformaldehyde and embedded in paraffin. The paraffin sections were deparaffinized to water, and the sections were put into xylene I, xylene II, absolute ethanol I, absolute ethanol II, 75% ethanol successively and washed with tap water; then stained with hematoxylin, differentiated with aqueous hydrochloric acid, turned blue with ammonia water solution and then washed. The sections were dehydrated with 85% and 95% gradient ethanol and stained in eosin staining solution, then put into anhydrous ethanol I, anhydrous ethanol II, anhydrous ethanol III, dimethyl I, xylene II successively, and the film was mounted with neutral gum. The pathological changes of the tissue were observed by an optical microscope (UOP, DSZ5000X, China). The histologic injury scores were graded on a scale of 0 to 4 as previously studied (22). Briefly, the severity of lung damage was rated using the following scoring system: 0: there is no harm and everything looks to be normal; 1, Minimal (damage of up to 25% of the field); 2, mild (injury of 25-50%of the field);3, moderate (injury of 50-75% of the field); and 4, severe (injury of >75% of the field). A pathologist who was unaware of the experiment examined tissue slices.

#### Lung Index

The lungs were collected and weighed, and the mice’s lung index was computed as follows:


Lung index(%)=lung weight (g)/body weight (g)×100

### Data Analysis

Each experiment was carried out at least three times. The statistical significance analysis was conducted using the GraphPad Prism 6.0 software (GraphPad Software, San Diego, CA). To compare groups, a one-way ANOVA with Dunnett’s multiple comparisons test was performed. Significant differences were defined as those with a P value less than 0.05 (P < 0.05).

## Results

### HSC Regulates LPS-Induced Pro-Inflammatory Transcription

**Figure 1:**
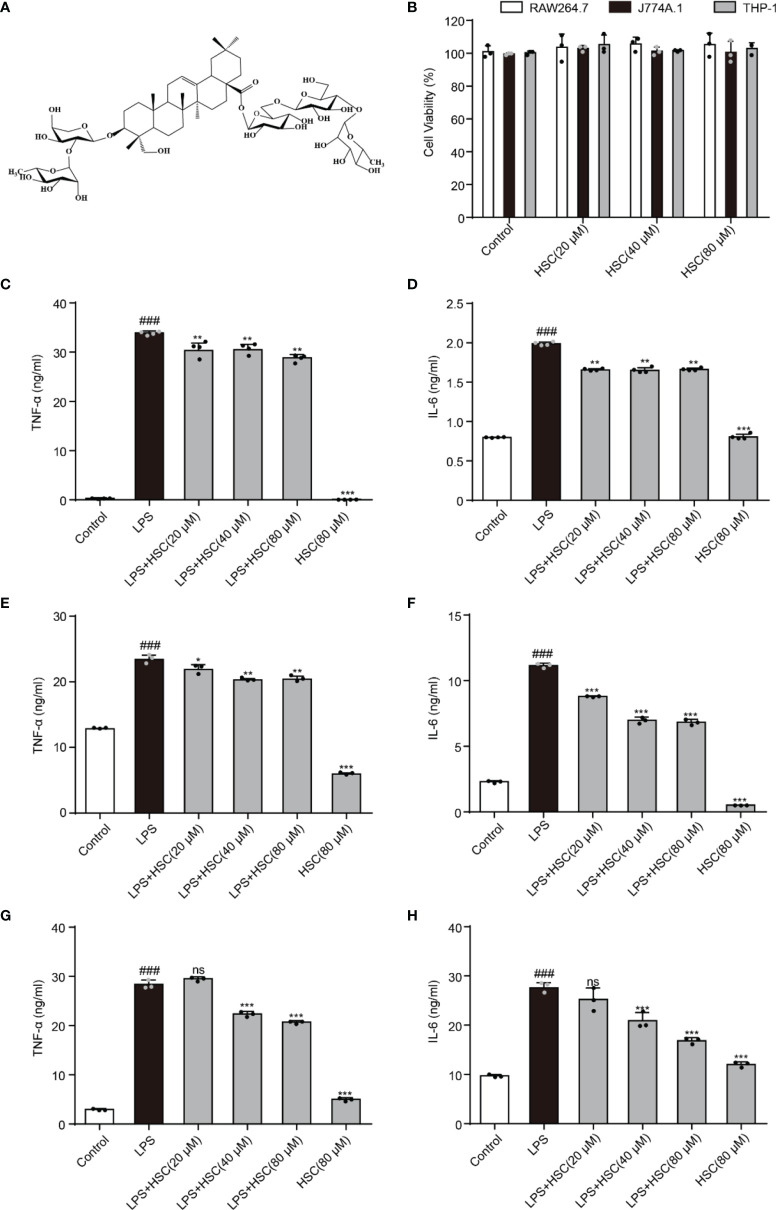
HSC regulates LPS-induced pro-inflammatory transcription. **(A)** The chemical structure of HSC; **(B)** RAW264.7, J774A.1, and THP-1cells were treated with HSC for 24 h, and cell viability was determined by the MTT assay; RAW264.7 **(C, D)**, J774A.1 **(E, F)**, and THP-1 **(G, H)** cells were treated with HSC for 4 h before co-incubation with LPS (1 μg/ml) for 18 h, the expression of TNF-α and IL-6 was detected using ELISA kits; ^###^ *P* < 0.001 compared to control alone group; *P < 0.05, **P < 0.01, and ***P < 0.001, compared to LPS alone group, ns, no significance (n=3).

As shown in **Figure 1A**, HSC is a triterpenoid saponin. Our findings showed that HSC (20, 40, and 80 μM) had no significant toxicity in RAW264.7, J774A.1, and THP-1 cells, respectively (**Figure 1B**). Epigenetic regulation is a key determinant of gene expression in sepsis (23). At the beginning of the infection, host-pathogen interactions usually lead to epigenetic changes in host cells that favor pathogen survival. At the same time, the host inflammatory response is characterized by epigenetic alterations in key regulatory genes, including TNF-a and IL-6 (24). TNF-α and IL-6 levels significantly increased in LPS-stimulated RAW 264.7 cells (*P*<0.001), while HSC (20, 40, and 80 μM) suppressed the level of TNF-α and IL-6 (Fig 1C and D) (F=1494, *P*<0.0001; F=1882, *P*<0.0001), which were further confirmed by J774A.1 (F=682.6, *P*<0.0001; F=2046, *P*<0.0001) and THP-1 cells (F=1756, *P*<0.0001; F=91.33, *P*<0.0001) (Fig 1E-H). Simultaneously, the qRT-PCR results indicated that HSC suppressed TNF-α and IL-6 at the gene expression level as well (F=555.6, *P*<0.0001; F=98.56, *P*<0.0001) (**Figure S1A**).

### Effects of HSC on the NF-κB Pathway

**Figure 2:**
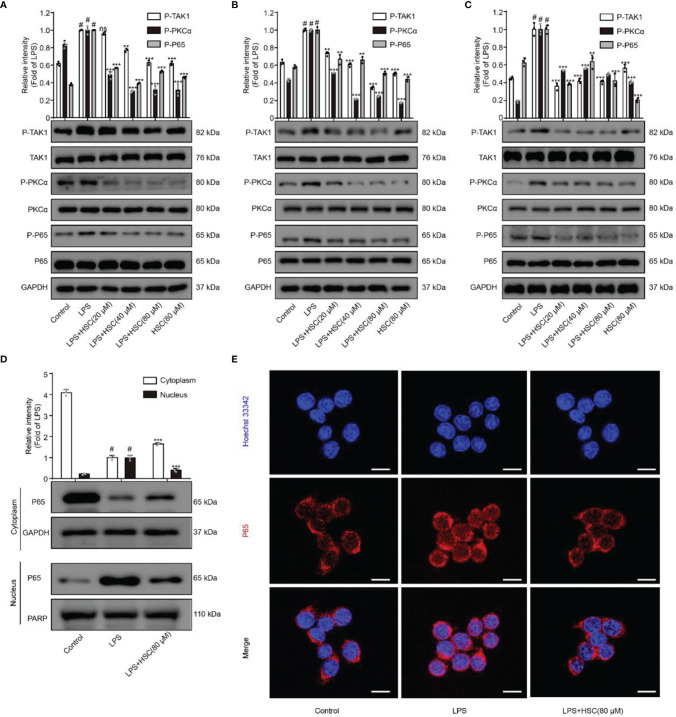
Effect of HSC on NF-κB pathway. RAW264.7, J774A.1, THP-1 cells were treated with HSC for 4 h then LPS (1 μg/mL) was added for 2 h. The protein expression of TAK1/PKC/p65 in RAW264.7 cell **(A)** J774A.1 cell **(B)** and THP-1 cell **(C)** by western blotting; **(D, E)** The localization of p65 in the cytoplasm and nucleus of RAW264.7 cells was detected by western blotting **(D)** and immunofluorescence **(E)** (Scale bar = 20 μm).; ^#^ *P* < 0.001 compared to control alone group; **P < 0.01, and ***P < 0.001, compared to LPS alone group, ns, no significance (n=3).

NF-κB, a transcription factor, is a heterodimer composed of p50 and p65, of which p65 is its most common form of transcriptionally activated component transcription factor (25), and has a crucial role in LPS-induced inflammation (26). The production of inflammatory mediators TNF-a and IL-6 as a result of LPS-induced nuclear translocation of NF-κB/p65 has been shown (27). The p-p65 protein expression was increased in LPS-stimulated macrophages, while pre-treatment with HSC dramatically reduced p-p65 protein expression (**Figures 2A–C**). Furthermore, LPS promoted the dissociation of NF-κB/p65 in the cytoplasm across the nuclear membrane into the nucleus in RAW264.7 cells, and HSC prevented NF-κB/p65 from translocation into the nucleus (**Figure 2D**), which was further confirmed by the immunofluorescence data (**Figure 2E**). Thus, we have concluded that HSC could reverse the LPS-induced nuclear translocation of NF-κB/p65.

NF-κB could be activated by multiple kinase signaling cascades and regulated by multiple factors (28). A previous study indicated that the protein kinase PKC could regulate the phosphorylation of p65 (29). Furthermore, Bing-C Chen et al. demonstrated that Ro 31-8220 (a PKC inhibitor) reduced the nuclear translocation of P65 that had been activated with LPS in J774A.1 cells (30), and Thu-Huyen Pham et al. also demonstrated that PKC is an upstream regulator of NF-κB (31). J. Wu et al. found that inhibition of p-TAK1 could further inhibit downstream activation of the signaling cascade, and thus NF-κB activation (32). As shown in **Figures 2A–C**, HSC significantly inhibited phosphorylation of TAK1 and PKCα in LPS-induced macrophages. Taken together, the results suggested that HSC inhibited NF-κB nuclear translocation in LPS-stimulated macrophages *via* the TAK1/PKCα pathway.

### HSC Inhibits the Activation of NLRP3 Inflammasome

**Figure 3:**
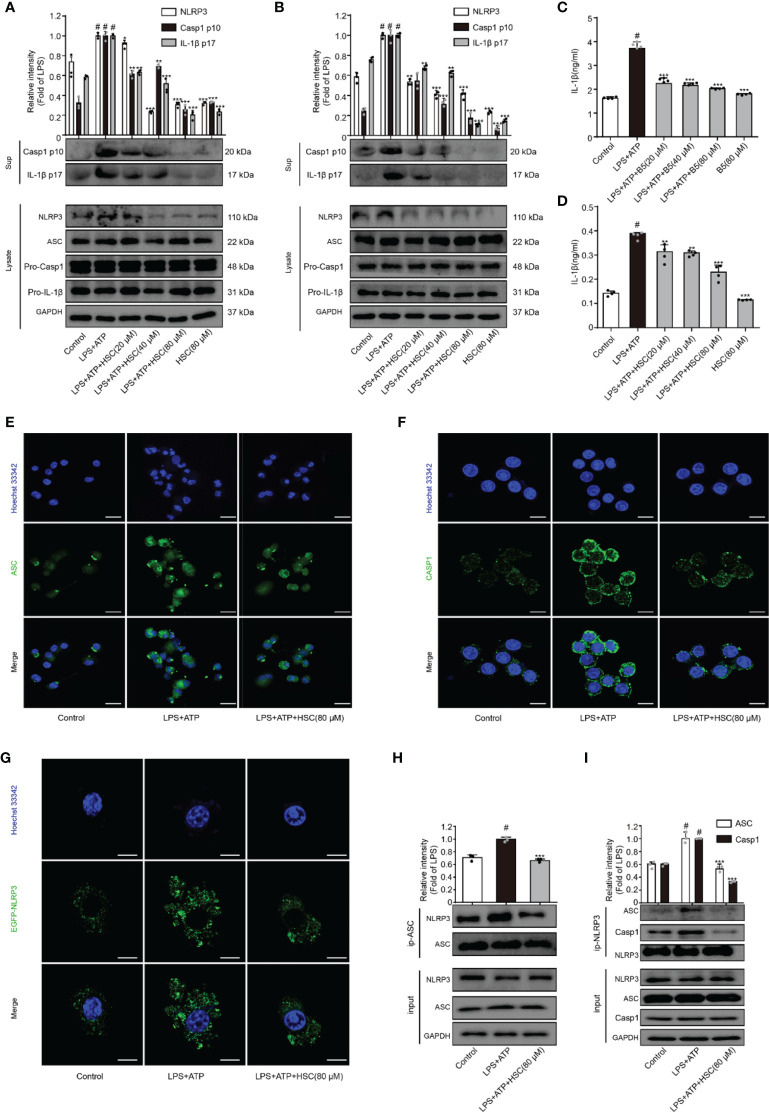
HSC inhibit the activation of NLRP3 inflammasome. J774A.1, THP-1 cells treated with HSC for 4 h, LPS (1 μg/mL) was added to stimulate for 6 h, followed by ATP (5 mM) co-culture for 30 min; **(A, B)** NLRP3 inflammasome related proteins NLRP3, ASC, caspase1, caspase-1p20, IL-1β, and IL-1β p17 were determined by western blotting in J774A.1 **(A)**, THP-1 **(B)** cells; **(C, D)** The level of IL-1β was determined by ELISA in J774A.1 **(C)**, THP-1**(D)**; **(E)** ASC activation was determined by immunofluorescence in THP-1 cells; **(F)** Cleaved-caspase-1 activation was determined by immunofluorescence in J774A.1 cells; **(G)** Plasmids of EGFP-NLRP3 were transfected into J774A.1 cells for 48 h, NLRP3 activation was determined by immunofluorescence; **(H)** The proteins were immunoprecipitated with ASC using magnetic beads, and the immunocomplexes were identified by western blotting. **(I)** Using magnetic beads, the obtained proteins were immunoprecipitated with NLRP3, and immunocomplexes were evaluated by western blotting; (Scale bar = 20 μm); ^#^ *P* < 0.001 compared to control alone group; ^*^ *P* < 0.05, ^**^ *P* < 0.01, and ^***^ *P* < 0.001, versus the LPS+ATP group, (n=3).

NLRP3 inflammasome plays a crucial role in inflammatory illnesses (33). To examine the effect of HSC on NLRP3 inflammasome, we investigated the NLRP3 inflammasome-associated proteins expression including NLRP3, ASC, caspase-1, and IL-1β. As shown in **Figures 3A, B**, LPS+ATP increased the expression of cleaved-caspase-1 and cleaved-IL-1β, which was significantly attenuated by HSC pretreatment. ELISA was used to determine the level of IL-1β in the supernatant, which is consistent with the protein expression (**Figures 3C, D**) (F=70.78, *P*<0.0001; F=117.8, *P*<0.0001). The inflammasome is a multi-protein complex that recruits pro-caspase-1 *via* ASC and then proceeds to cleave the cytokine precursors pro-IL-1β and pro-IL-18 into mature IL-1β and IL-18 (34). As shown in **Figures 3A, B**, LPS+ATP exerted no effects on the expression of ASC and caspase-1. However, immunofluorescence results showed a significant increase in ASC spots (green spots) in LPS+ATP-stimulated THP-1 cells, and the ASC spot (green spot) formation was decreased by HSC (80 μM) (**Figure 3E**). The immunofluorescence results showed that LPS+ATP-triggered J774A.1 cells activated caspase-1 expression, while the activation of caspase-1 was suppressed by HSC (**Figure 3F**).

A two-signal model for how the NLRP3 inflammasome is primed. It could be caused by microbial components or endogenous cytokines to activate the transcription factor NF-κB, resulting in the up-regulation of NLRP3 and pro-IL-1β. The activation signal comes from a variety of factors like extracellular ATP, RNA viruses, and particulate matter. Multiple molecular or the assembly of NLRP3, ASC, and caspase-1 into inflammasome to promote pro-IL-1β uncapping into IL-1β release into the extracellular (35). LPS+ATP increased the expression of NLRP3, which was significantly attenuated by HSC (**Figures 3A, B**). J774A.1 cells were transfected with an EGFP-NLRP3 plasmid. Immunofluorescence intensity showed the same results as western blotting (**Figure 3G**). Using Co-IP assays, we found that HSC significantly decreased the formation of NLRP3 and ASC complexes when LPS+ATP was added to the cells (**Figure 3H**). HSC also dramatically decreased the formation of NLRP3, ASC, caspase-1 complexes (**Figure 3I**). Thus, these results indicated that HSC could inhibit NLRP3 inflammasome activation.

### Effect of HSC on Ca^2+^ Release in Macrophages

**Figure 4:**
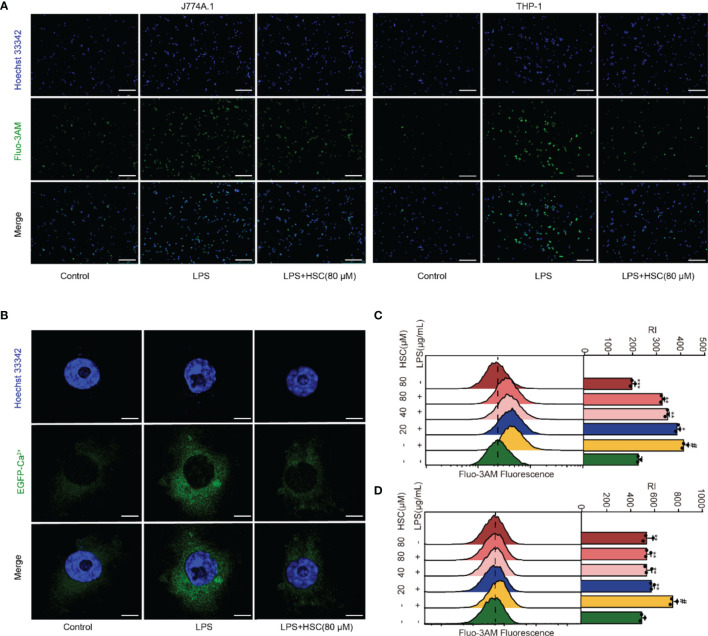
Effect of HSC on Ca^2+^ release in macrophages. RAW264.7, J774A.1, THP-1 cells were treated with HSC for 4 h and incubated with LPS for 6 h; **(A)** J774A.1 and THP-1 cells were stained with Fluo-3/AM (10 μM) for 1 h. The images were captured by fluorescence microscopy (Scale bar=10 μm); **(B)** Plasmid of EGFP-Ca^2+^ was transfected in J774A.1 cells for 48 h, Ca^2+^ fluorescence was determined by immunofluorescence (Scale bar=20 μm); **(C, D)** RAW264.7 and THP-1 cells were stained with Fluo-3/AM for 1 h, and the fluorescence intensity was assessed by flow cytometry; ^#^ *P* < 0.001 compared to control alone group; ^*^ *P* < 0.05, ^**^ *P* < 0.01, and ^***^ *P* <0 .001, versus the LPS group, (n=3).

Ca^2+^ has an irreplaceable regulatory role in many cell functions and is a vital signal factor in the inflammatory response (36). The Ca^2+^ release was detected by using immunofluorescent, the level of Ca^2+^ significantly increased in LPS-treated cells, while HSC suppressed the Ca^2+^ release (**Figure 4A**). As shown in **Figures 4C, D**, the flow cytometry analysis revealed that HSC decreased Ca^2+^ level, which was further confirmed by J774A.1 cells transfected with an EGFP-Ca^2+^ plasmid (**Figure 4B**). Taken together, the results indicated that HSC inhibited LPS-stimulated Ca^2+^ release.

### Effect of HSC on the PIP2 Signaling Pathway

**Figure 5:**
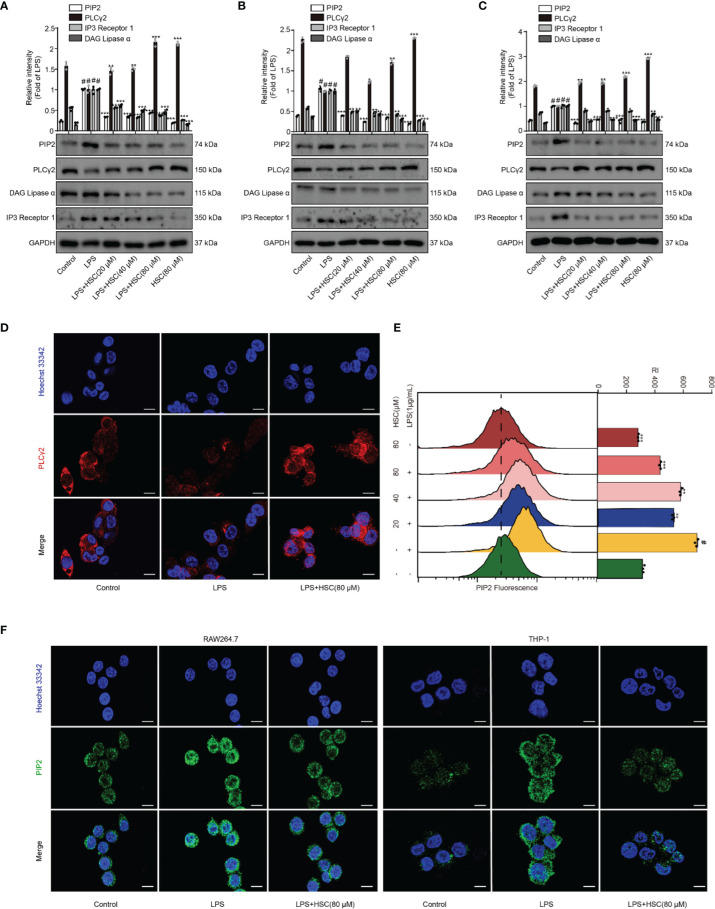
Effect of HSC on the PIP2 signaling pathway. **(A)** RAW264.7, J774A.1, THP-1 cells treated with HSC for 4 h, and then induced by LPS (1 μg/ml) for 1 h; (A, B, and C) The expression of PIP2, PLCγ2, DAG, and IP3 were detected by western blotting in RAW264.7 **(A)**, J774A.1 **(B)**, THP-1 **(C)** cells; **(D)** The PLCγ2 expression was detected by immunofluorescence assay in RAW264.7 cells; **(E)** Detection of PIP2 expression in RAW264.7 cells by flow cytometry; **(F)** Detection of PIP2 expression by immunofluorescence in RAW264.7 and THP-1 cells (Scale bar=20 μm); ^#^ *P* < 0.001 compared to control alone group; ^*^ *P* < 0.05, ^**^ *P* < 0.01, and ^***^ *P* < 0.001, versus the LPS group, (n=3).

PLC degrades PIP2 lipids and regulates multiple cellular events (12). Activation of PKC and release of intracellular calcium from the endoplasmic reticulum are mediated by DAG and IP3, which are produced by PLCγ2 catalyzing PIP2 (37), leading to the activation of the NF-κB and NLRP3 inflammasome pathways (16, 38). As shown in **Figure 5A**, LPS stimulation up-regulated PIP2, IP3, and DAG expression, while pretreatment with HSC slightly down-regulated PIP2, IP3, and DAG protein expression. Pretreatment with HSC increased PLCγ2 protein expression in RAW264.7 cells. It was further verified in J774A.1 and THP-1 cells (**Figures 5B, C**). Meanwhile, the qRT-PCR study indicated that PIP2, PLCγ2, DAG, and IP3 were obtained similar at the gene expression level in LPS-induced J774A.1 cells (F=63.25, *P*<0.0001; F=660.8, *P*<0.0001; F=129.1, *P*<0.0001; F=40.13, *P*=0.0003) (**Figure S1B**). Immunofluorescence results showed that PLCγ2 fluorescence (red) was significantly diminished under LPS-induced RAW264.7 cells, while red fluorescence was stronger in the HSC group, suggesting that HSC could promote PLCγ2 expression (**Figure 5D**). We also further examined the effect on PIP2 expression by using flow cytometry and immunofluorescence. As shown in **Figure 5E**, LPS-stimulated PIP2 expression in RAW264.7 cells was increased, while HSC inhibited PIP2 expression. The immunofluorescence results showed that PIP2 fluorescence (green) was significantly increased in LPS-stimulated RAW264.7 and THP-1 cells, while the green fluorescence was weaker in the HSC group (**Figure 5F**).

### HSC Inhibits the Activation of TAK1 *via* PIP2

**Figure 6:**
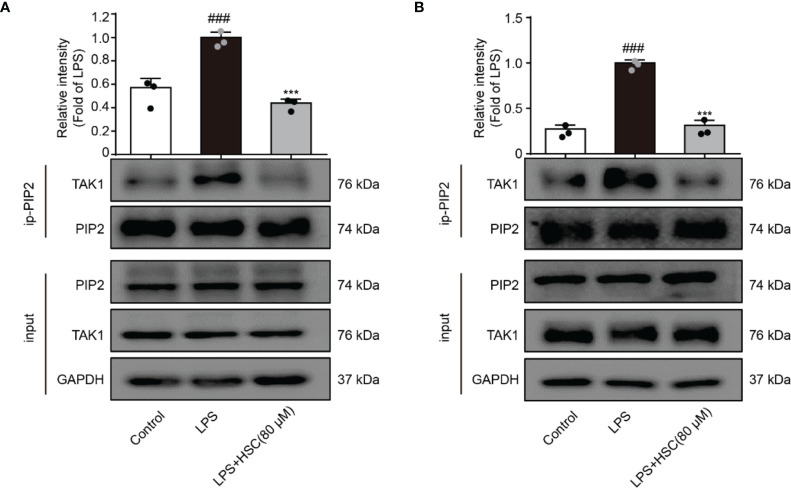
HSC inhibits the activation of TAK1 *via* PIP2. RAW264.7 **(A)**, THP-1 **(B)** cells treated with HSC for 4 h, and then induced by LPS (1 μg/ml) for 1 h; The collected proteins were immunoprecipitated with PIP2 using magnetic beads, and immunocomplexes were determined by western blotting; ^###^ *P* < 0.001 compared to control alone group; ^***^ *P* < 0.001 versus the LPS group, (n=3).

TAK1, a highly conserved MAPK kinase, is a key regulator in the innate immune response (39). PIP2 lipids bind directly to TAK1 at W241 and N245. TAK1 activation and the associated generation of pro-inflammatory cytokines are eliminated when PIP2’s binding affinity is impaired or the PIP2 binding site is mutated (12). As shown in **Figures 6A, B**, LPS-stimulated RAW264.7 and THP-1 cells increased interaction of endogenous TAK1 with endogenous PIP2, while HSC attenuated the combination between TAK1 and PIP2.

### HSC Protects LPS-Induced ALI and Improves Respiratory Function

**Figure 7:**
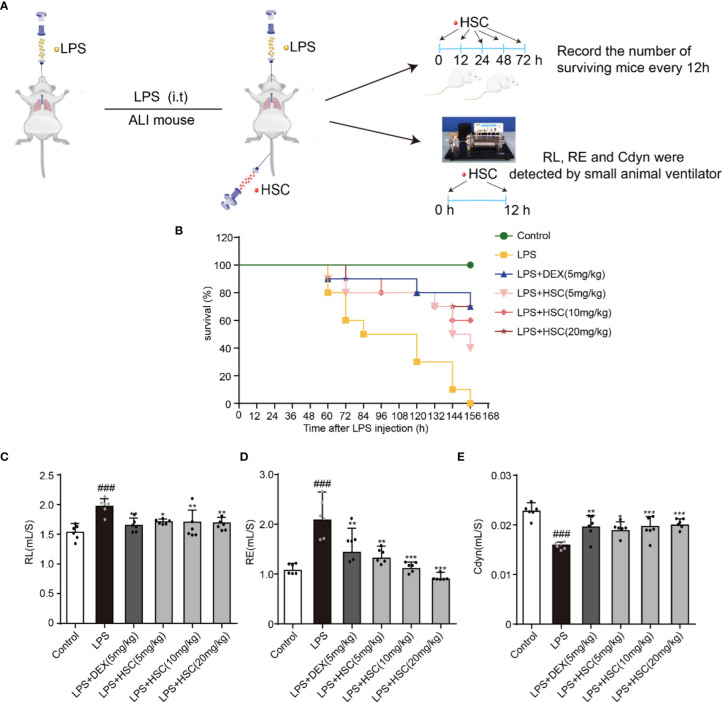
HSC protected LPS-induced ALI and improves respiratory function. **(A)** The mice were treated with LPS (15 mg/kg, i.t.), and treated with HSC (5, 10, and 20 mg/kg, i.v.) for 0, 12, 24, 48, and 72 h. Dexamethasone (DEX; 5 mg/kg, i.p.) was used as a positive control. **(B)**The survival rates of all the mice were observed for a period of 168 h, (n=10). **(C-E)** The mice were treated with LPS (4 mg/kg, i.t.). After LPS injection, mice were treated with HSC at 0 and 12 h. After 24h, blood samples were collected, neutrophils, **(C)** Resistance of lung (RL), **(D)** resistance of expiration (Re), and **(E)** respiratory lung compliance (Cdyn) levels were detected using an AniRes2005 lung function test system, (n=6). ^###^ *P* < 0.001 compared to control alone group; ^*^ *P* < 0.05, ^**^ *P* < 0.01, and ^***^ *P* < 0.001 versus the LPS group.

In this study, the sepsis model was established to investigate the effect of HSC on ALI. Intratracheal non-invasive instillation of LPS (15 mg/kg, i.t.) was used to successfully establish a sepsis model (**Figure 7A**). In the LPS-induced sepsis group, all mice died within 156 h. However, HSC (5, 10, and 20 mg/kg) rescued the mice’s survival rate (40%, 60%, and 70%) in 156 h (**Figure 7B**), of which the effect was similar to that of the DEX, a positive drug (70%). Furthermore, respiratory mechanics research revealed that the resistance of the lung (RL) and resistance of expiration (Re) were increased in the LPS group, while they were significantly decreased in the HSC-treated group (**Figures 7C, D**) (F=7.321, *P*<0.0001; F=10.21, *P*<0.0001). HSC significantly increased respiratory lung compliance (Cdyn) level (**Figure 7E**) (F=11.78, *P*<0.0001).

### HSC Ameliorates LPS-Induced ALI in Mice

**Figure 8:**
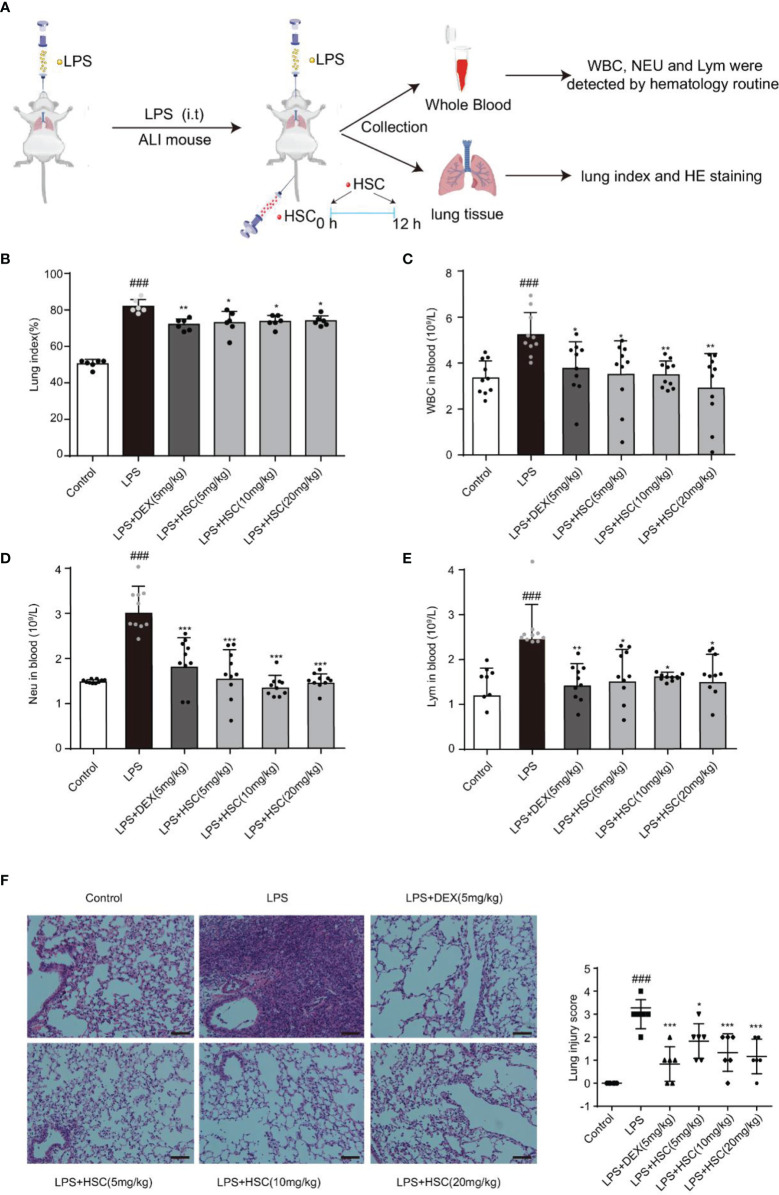
HSC ameliorated LPS-induced ALI in mice. **(A)** The mice were treated with LPS (4 mg/kg, i.t.). After LPS injection, mice were treated with HSC at 0 and12 h, and the corresponding indexes were measured 24 h later. **(B)** Lung index (n=6); **(C)** White blood cells (WBC), **(D)** neutrophil and **(E)** lymphocyte counts in the blood, which were determined by an auto hematology analyzer (n=10), **(F)** Lung histopathology was assessed *via* H&E staining assay at 24 h after LPS challenge (200×) (n=6); ^###^ *P* < 0.001 compared to control alone group; ^*^ *P* < 0.05, ^**^ *P* < 0.01, and ^***^ *P* < 0.001 versus the LPS group.

To validate HSC’s therapeutic effects on ALI, mice were treated with LPS by non-invasive intratracheal instillation, and the lung index, white blood cells (WBC), neutrophil, and lymphocyte were measured, along with hematoxylin and eosin (H&E) (**Figure 8A**). The findings suggested that LPS increased the lung index and caused damage and enlargement of the lung, which was considerably reduced by the treatment with HSC (**Figure 8B**) (F=61.15, *P*<0.0001). The excessive rise in white blood cells (WBC), neutrophils, and lymphocyte levels caused by LPS was dramatically reduced after treatment with HSC (**Figures 8C–E**) (F=5.085, *P*=0.0007; F=17.42, *P*<0.0001; F=5.098, *P*=0.0007). And the body weight was not affected by HSC compared to the LPS group (**Figures S2A, B**) (F=28.56, *P*>0.05). Additionally, H&E staining results revealed that LPS induced alveolar interstitial exudation disrupted the normal structure of alveoli, and resulted in the infiltration of a high number of inflammatory cells. HSC, as well as the DEX, ameliorated lung tissue damages (**Figure 8F**).

### HSC Regulates the Inflammatory Process of ALI

**Figure 9:**
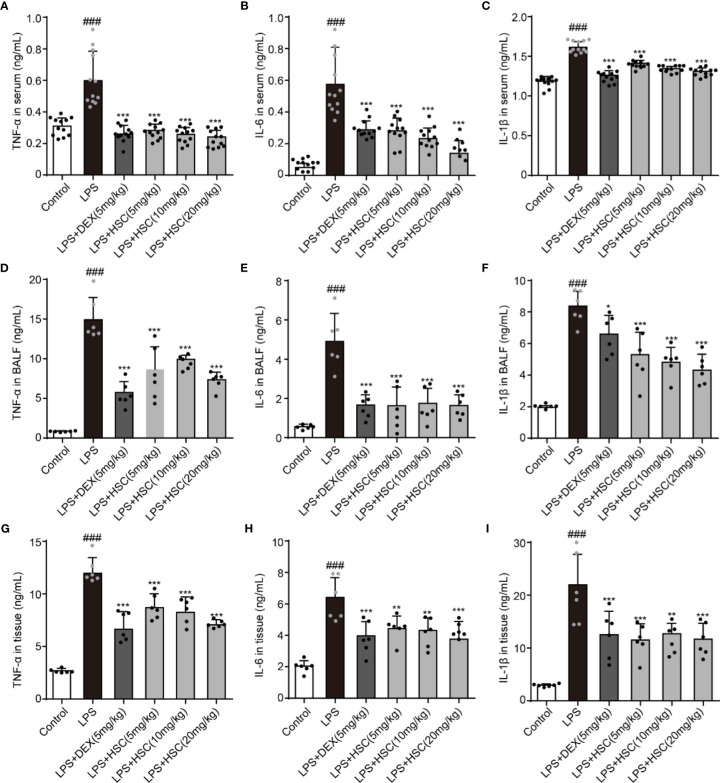
HSC regulates the inflammatory process of ALI. The mice were treated with LPS (4 mg/kg, i.t.). After LPS injection, mice were treated with HSC at 0 and 12 h, and the corresponding indexes were measured 24 h later. **(A–C)** The serum of mice was collected to determine the inflammatory cytokines TNF-α, IL-1β and IL-6 were detected by ELISA (n=12); **(D–F)** The BALF of mice were collected to determine the inflammatory cytokines TNF-α, IL-1β, and IL-6 were detected by ELISA (n=6); **(G–I)** The lung tissue of mice were collected to determine the inflammatory cytokines TNF-α, IL-1β and IL-6 were detected by ELISA (n=6). ^###^ *P* < 0.001 compared to control alone group; ^*^ *P* < 0.05, ^**^ *P* < 0.01, and ^***^ *P* < 0.001 versus the LPS group.

LPS-induced lung inflammation is a typical form of ALI with the production of inflammatory cytokines such as TNF-α, IL-1β, and IL-6 (40). To test the effect of HSC on the inflammatory response in ALI, the serum, BALF, and lung tissues were collected, and the level of inflammatory response was determined. Our results indicated that LPS increased the level of TNF-α, IL-1β, and IL-6 in serum, BALF, and lung tissues, while DEX and HSC decreased the level of TNF-α, IL-6, and IL-1β in serum (**Figures 9A–C**) (F=50.84, *P*<0.0001; F=34.24, *P*<0.0001; F=94.84, *P*<0.0001), TNF-α, IL-6, and IL-1β in BALF (**Figures 9D–F**) (F=37.68, *P*<0.0001; F=26.37, *P*<0.0001; F=31.71, *P*<0.0001), and TNF-α, IL-6, and IL-1β in lung tissues (**Figures 9G–I**) (F=36.24, *P*<0.0001; F=23.57, *P*<0.0001; F=16.07, *P*<0.0001). These results demonstrated that HSC had a significant anti-inflammatory effect on ALI.

### HSC Ameliorates ALI in Mice by Regulating the PIP2/NF-κB/NLRP3 Pathway

**Figure 10:**
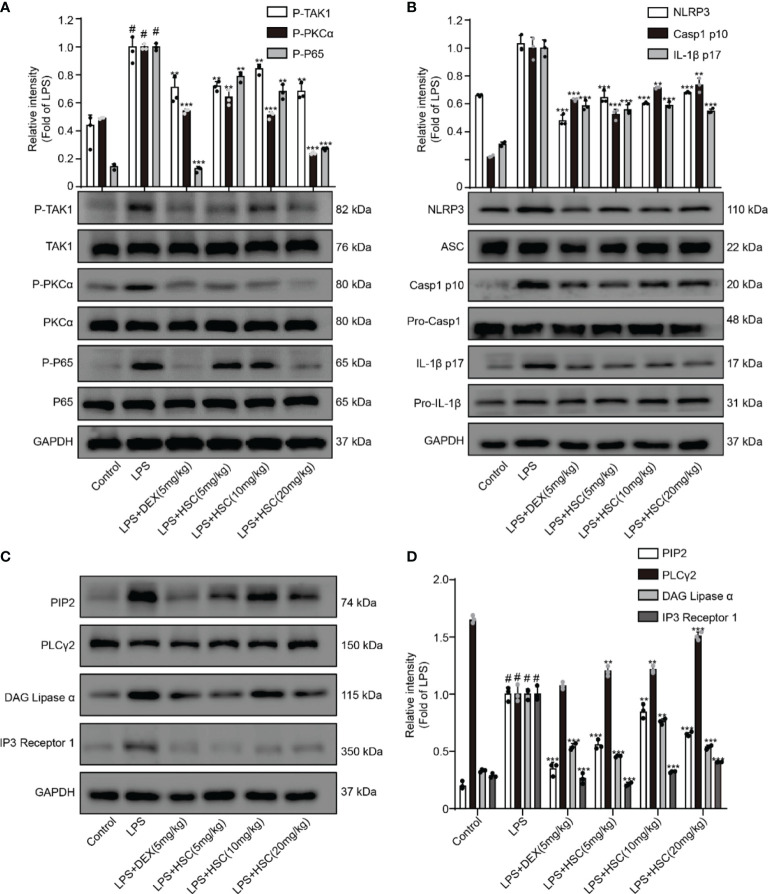
HSC ameliorates ALI in mice by regulating the NF-κB/PIP2/NLRP3 pathway. The mice were treated with LPS (4 mg/kg, i.t.). After LPS injection, mice were treated with HSC at 0 and 12 h, and the corresponding indexes were measured 24 h later. **(A)** Detection of TAK1/P-TAK1, PKCα/p-PKCα and p65/p-p65 protein expression in lung tissue by western blotting; **(B)** Detection of NLRP3/ASC/pro-caspa1/Caspa1 p10/IL-1β/IL-1β p17 protein expression in lung tissue; **(C)** PIP2/PLCγ2/DAG/IP3 protein expression in lung tissue by western blotting, and **(D)** statistical analysis of PIP2/PLCγ2/DAG/IP3 protein; ^###^ *P* < 0.001 compared to control alone group; ^**^ *P* < 0.01, and ^***^ *P* < 0.001 versus the LPS group (n=6).

A recent study shows NF-κB has a critical role in ALI formation and progression (41). LPS stimulates the NF-κB signaling pathway in lung tissue, resulting in the release of pro-inflammatory cytokines such as IL-1β, IL-6, and TNF-α in lung tissue (42). Our results showed that the phosphorylation of TAK1, PKCα, and p65 in lung tissue was significantly increased by LPS, whereas phosphorylation of TAK1, PKCα, and p65 was significantly decreased by DEX and HSC. (**Figure 10A**).

A previous study revealed that the participation of the NLRP3 inflammasome is required for the development of ALI (43). When the NLRP3 inflammasome was activated, cleaved-caspase-1 and cleaved-IL-1β were over-expressed, leading to neutrophil recruitment, which in turn promotes the development of ALI (44). As shown in **Figure 10B**, LPS increased the expression of NLRP3, cleaved-caspase-1, and cleaved-IL-1β, all of which were reversed by treatment with HSC and DEX. The expression of PIP2, DAG, and IP3 proteins was up-regulated and PLCγ2 protein expression was down-regulated by LPS stimulated, while DEX and HSC significantly reversed the expression of these proteins (**Figures 10C, D**).

## Discussion

ALI is a disease that loses lung function and has a high death rate, which is caused by an uncontrolled inflammatory response. The study has been shown that the pathogenesis of ALI is summarized as the early alveolar inflammation accompanying lung injury (45, 46). Gram-negative bacteria produce large amounts of LPS, which is a significant component of the bacterium, causes a strong immune response in animals, and is used to induce ALI/ARDS (47). HSC, a natural product isolated from the plant *Pulsatilla chinensis* (Bunge), has a wide range of biological activity (19). However, the precise mechanism of HSC’s anti-inflammatory action, as well as its therapeutic effect on ALI, has not yet been illustrated clearly. In this study, it was determined that HSC had anti-inflammatory activity in LPS-induced macrophages, that HSC had a therapeutic effect in LPS-induced ALI.

Under the action of pathogenic microorganisms, it activates the target cells in the lung and promotes the cells to secrete inflammatory mediators likes TNF-α, IL-6, and IL-1β. These cytokines cause endothelial cells to become more active and cause edema in the lung, which can be life-threatening (48). TNF-α and IL-6 are important cytokines in the development of ALI, which can activate the endothelial cells and lead to pulmonary edema (49). Macrophages release the active cytokine IL-1β, which is increased in serum, BALF, and lung tissue of ALI. Persistent and increasing IL-1β levels are associated with poor prognosis (7). In addition, neutrophils, lymphocytes, and leukocytes play an irreplaceable role in mediating inflammatory and immune responses, which not only kill invading pathogenic microorganisms but also exacerbate tissue and organ damage (50). Therefore, seeking drugs that can reduce inflammatory cytokines, neutrophils, lymphocytes, and leukocytes is an effective strategy of the treatment with ALI. Our findings revealed that HSC could greatly suppress the production of TNF-α, IL-6 and IL-1β in macrophages cells stimulated by LPS/LPS+ATP. In addition, HSC has a therapeutic effect on ALI through decreasing the concentration of neutrophils, lymphocytes and leukocytes, inhibiting the inflammatory cytokines production such as TNF-α, IL-1β, and IL-6, and ameliorating the lesion of lung tissues and the inflammation infiltration in the alveolar.

The NF-κB signaling pathway has an irreplaceable role in regulating excessive inflammatory damage and response (51, 52). When the NF-κB signaling pathway is engaged, inflammatory factors like TNF-α, IL-6, and IL-1β are generated during ALI (53, 54). TAK1 is a required kinase for TLR4-mediated NF-κB activation (32). PKC inhibitor Ro 31-8220 inhibits LPS-stimulated NF-κB activation, indicating that PKC is a complementary route for LPS-mediated NF-κB activation (30). Our findings showed that HSC significantly decreased LPS-induced phosphorylation of TAK1, PKCα, and p65, as well as p65 nuclear translocation in LPS-stimulated macrophages and-induced ALI mice. Collectively, the NF-κB pathway participates in the therapeutic effect of HSC on LPS-induced ALI.

In LPS+ATP-stimulated macrophage cells, the intracellular Ca^2+^ balance is disrupted, which promotes the activation of NLRP3 inflammasome (55). When the NLRP3 inflammasome is activated, the production of cleaved caspase-1 and mature IL-1β is increased, resulting in the neutrophil recruitment to develop ALI (35). As a result, suppression of NLRP3 inflammasome activation offers an alternate method for ALI therapy. Our finding revealed that HSC had an anti-inflammatory activity by preventing the activation of NLRP3 inflammasomes and inhibiting the expression of NLRP3, cleaved-caspase-1, and cleaved-IL-1β and the release of IL-1β. In addition, HSC also inhibited NLRP3, cleaved caspase-1, and cleaved IL-1β expression in the lung tissue of LPS-induced ALI mice.

Acute lung injury is characterized by epigenetic instability, one of its most prominent characteristics, which is involved in altered chromatin remodeling, dysregulation of non-coding RNA (ncRNA), histone modifications, and altered DNA methylation status (56). These modifications alter gene expression, replication, and DNA repair. Phosphatidylinositol is one of the crucial factors in inflammation development. It can also induce various types of DNA damage and modifications in DNA structure (9). PIP2 works as a transcription activator by interfering with the capacity of both RNA polymerases I and II to bind DNA to change the chromatin structure (57). PLCγ2, one of the key enzymes in the phosphoinositide metabolism system, hydrolyzes PIP2 to generate two-second messengers, inositol IP3 and DAG. DAG mediates the activation of PKC and IP3 triggers the release of Ca^2+^ in the cells (9, 58). PIP2 inhibits the activation of TKA1 by directly interacting with TAK1, to inhibit the production of pro-inflammatory cytokines (12). In this study, our data indicated that HSC inhibited the expression of PIP2, IP3, DAG, and promoted the expression of PLCγ2. The immunofluorescence and flow cytometry analysis indicated that HSC inhibited the release of Ca^2+^ in macrophages stimulated by LPS. In LPS-induced ALI, HSC suppressed PIP2, IP3, and DAG expression, and enhanced PLCγ2 expression. Taken together, these results suggested HSC reduced the calcium level *via* PIP2 signaling pathway, which subsequently inhibited the activation of NLRP3 inflammasome, and protected LPS-ALI mice.

**Figure 11:**
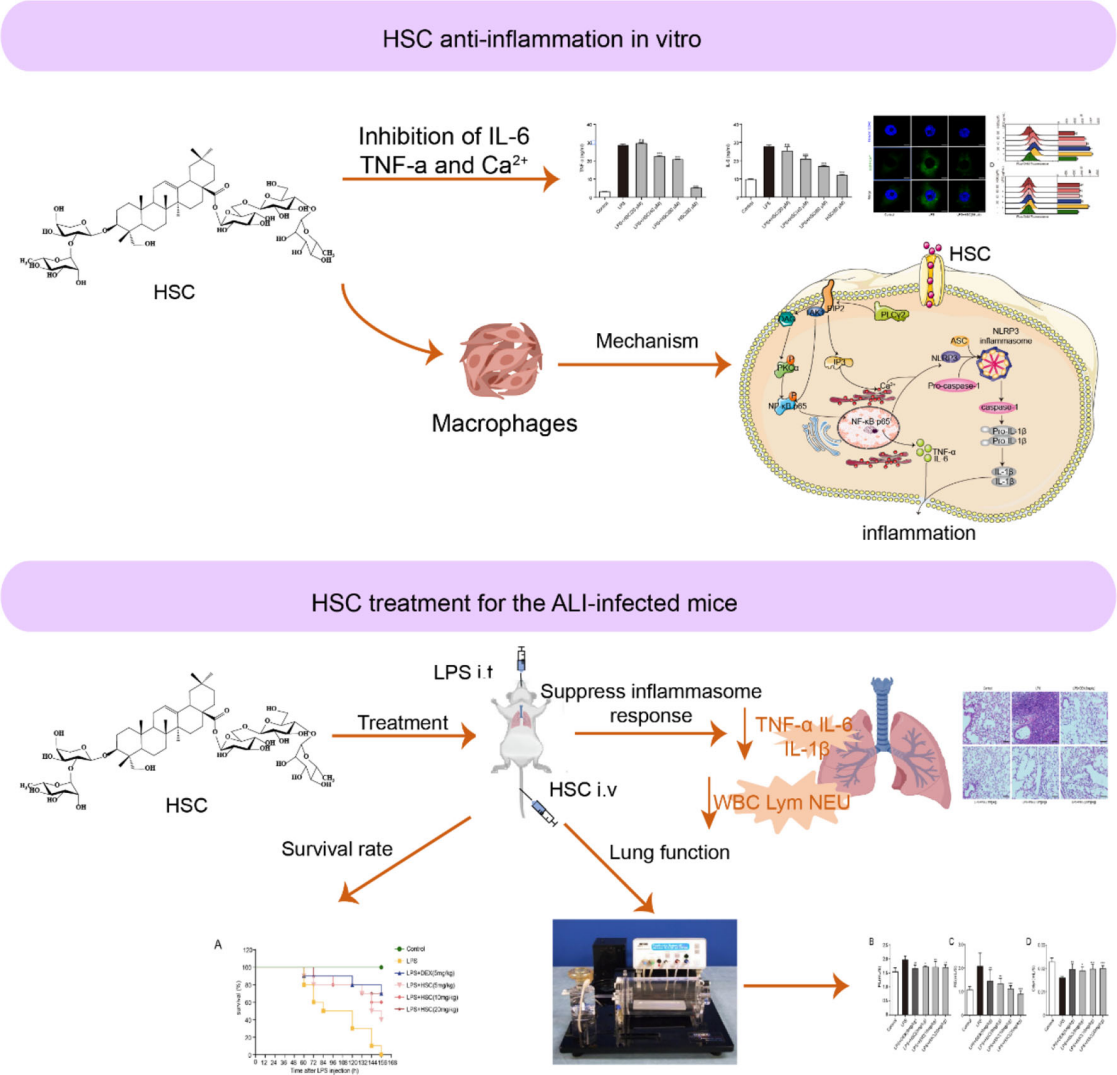
Working model for the HSC-based therapeutic effect of ALI.

In conclusion, we found that HSC, a triterpenoid saponin, inhibited PIP2, an epigenetic regulator, to exert a therapeutic effect on ALI. HSC significantly suppressed cytokines storm like IL-6, IL-1β, and TNF-α through the NF-κB pathway, the NLRP3 inflammasome, and intracellular Ca^2+^-related signaling pathways, all of which were affected by PIP2 (**Figure 11**). Thus, HSC might target PIP2 to exert an anti-inflammatory effect *in vitro* and *in vivo*. However, the detailed target is remaining to be further studied. Of interest, HSC inhibited pro-inflammatory cytokines storm and ameliorated the injury of lung tissue, of which the phenomenon is similar to a patient infected by SARS-CoV-2, suggesting that HSC might be a potential agent to treat with COVID-19, which is deserved to study in further study.

## Data Availability Statement

The original contributions presented in the study are included in the article/**Supplementary Material**. Further inquiries can be directed to the corresponding author.

## Ethics Statement

The animal study was reviewed and approved by Guangxi University of Chinese Medicine (approval document number: SYXK-GUI-2019-0001).

## Author Contributions

HG designed the research. SH and RY conducted chemical experiments and part of anti-inflammatory experiments. SH, Q-QW, and YZ conducted experiments in vivo. SH, YC, and JH wrote the manuscript. SY and HG revised the manuscript. All authors contributed to the article and approved the submitted version.

## Conflict of Interest

The authors declare that the research was conducted in the absence of any commercial or financial relationships that could be construed as a potential conflict of interest.

## Publisher’s Note

All claims expressed in this article are solely those of the authors and do not necessarily represent those of their affiliated organizations, or those of the publisher, the editors and the reviewers. Any product that may be evaluated in this article, or claim that may be made by its manufacturer, is not guaranteed or endorsed by the publisher.

## Abbreviations

ALI, acute lung injury; ASC, apoptosis-associated speck-like protein containing a CARD; ATP, adenosine triphosphate; BALF, bronchoalveolar lavage fluid; DMEM, Dulbecco’s modified eagle medium; DAG, diacylglycerol; DEX, dexamethasone; ELISA, Enzyme-linked immunosorbent assay; H&E, hematoxylin and eosin; IP, immunoprecipitation; IL-1β, interleukin-1β; IL-6, interleukin-6; LPS, lipopolysaccharide; NLRP3, nod-like receptor 3; NF-κB, nuclear factor-κB; PLC, phospholipase C; PIP2, phosphatidylinositol 4,5-bisphosphate; rRNA, ribosomal ribonucleic acid; TNF-α, tumor necrosis factor-α; TAK1, transforming growth factor-β activated kinase 1; WBC, white blood cell.
