# Supplementary material for: Hederasaponin C Alleviates Lipopolysaccharide-Induced Acute Lung Injury In Vivo and In Vitro Through the PIP2/NF-κB/NLRP3 Signaling Pathway
Source: Front Immunol. 2022 Feb 25;13:846384. doi: 10.3389/fimmu.2022.846384 (PMC8913935; doi:10.3389/fimmu.2022.846384)
Supplement: Supplementary file 1 [file DataSheet_1.docx]

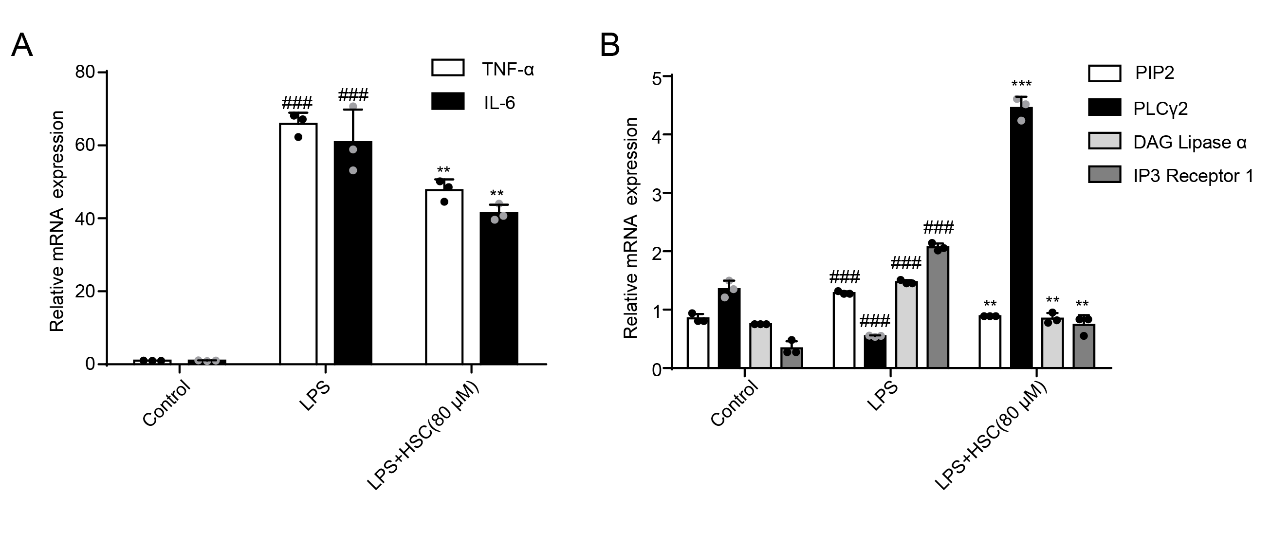


Fig S1. (A) J774A.1 cells treated with HSC (80 μM) for 4 h, and then induced by LPS (1 μg/ml) for 6 h, the expression of TNF-α and IL-6 were measured by qRT-PCR. (B) J774A.1 cells treated with HSC (80 μM) for 4 h, and then induced by LPS (1 μg/ml) for 1 h, the expression of PIP2, PLCγ2, DAG, and IP3 were measured by qRT-PCR.


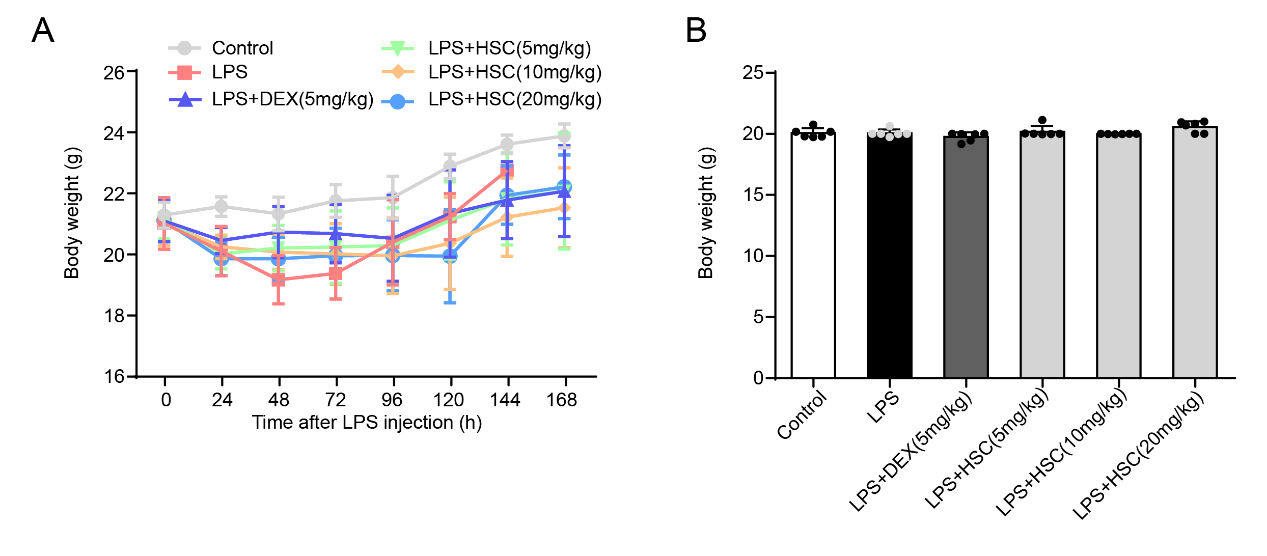


Fig S2. The mice were treated with LPS (4 mg/kg, i.t.). (A) The mice were treated with LPS (15 mg/kg, i.t.), and treated with HSC for 0, 12, 24, 48, and 72 h. Dexamethasone (DEX; 5 mg/kg, i.p.) was used as a positive control. The effect of HSC on mice body weight (n=10); (B) After LPS (4 mg/kg, i.t.) injection, mice were treated with HSC at 0, 12 h, and the corresponding indexes were measured 24 h later. The effect of HSC on mice body weight (n=6).
